# Do non-drinking youth drink less alcohol in young adulthood or do they catch up? Findings from a Swedish birth cohort

**DOI:** 10.1093/eurpub/ckad057

**Published:** 2023-04-20

**Authors:** Peter Larm, Charlotta Hellström, Jonas Raninen, Cecilia Åslund, Kent W Nilsson, Fabrizia Giannotta

**Affiliations:** Department of Public Health Sciences, Stockholm University, Stockholm, Sweden; School of Health, Care and Social Welfare, Mälardalen University, Västerås, Sweden; Department of Clinical Neuroscience, Karolinska Institutet, Stockholm, Sweden; Centre for Clinical Research, Uppsala University, Västmanland County Hospital Västerås, Västerås, Sweden; Department of Public Health and Caring Sciences, Uppsala University, Uppsala, Sweden; School of Health, Care and Social Welfare, Mälardalen University, Västerås, Sweden; Centre for Clinical Research, Uppsala University, Västmanland County Hospital Västerås, Västerås, Sweden; Department of Public Health Sciences, Stockholm University, Stockholm, Sweden

## Abstract

**Background:**

Alcohol consumption among adolescents has declined considerably during the last two decades. However, it is unknown if these adolescents’ alcohol consumption will remain low as they grow older. To our knowledge, this is one of the first studies that uses longitudinal data to examine if non-drinking adolescents have a lower alcohol consumption in young adulthood or if they catch up.

**Methods:**

A self-report survey was distributed to a birth cohort (*n* = 794) born in 1997 in a Swedish region when cohort members attended ninth grade (age 14–15 years) in 2012. Responders were divided into non-drinkers and alcohol users and assessed again in their late teens (17–18 years) and young adulthood (20–21 years).

**Results:**

In their late teens (17–18 years), non-drinkers at baseline consumed less alcohol and had a lower probability of harmful use compared with their alcohol-using peers. In young adulthood (20–21 years), these effects disappeared when adjustment was made for covariates. However, a stratified analysis showed that non-drinking adolescents low in conduct problems consumed less alcohol and had a lower probability of harmful use in young adulthood than alcohol-using peers.

**Conclusions:**

This study suggests that the decline in alcohol use among adolescents in the past decades may be associated with a lower alcohol consumption in the late teens and young adulthood among those low in conduct problems. This may have promising implications for alcohol-related morbidity and mortality.

## Introduction

During the last two decades, a major decline in adolescents’ alcohol consumption has occurred, in particular in English-speaking countries, such as the USA, the UK and Australia, as well as in the Nordic countries (except Denmark).^1^^,^^2^ In Sweden, the proportion of non-drinking ninth graders (15–16 years) has increased from 22% in 1999 to 58% in 2019.^3^ Although research has focused on why this trend has occurred, evidence remains scarce.^4^ The increased trend of non-drinking adolescents has pushed the age of alcohol onset upwards,^5^^,^^6^ which may have great public health benefits given that some studies have shown an early age of onset to increase the risk of subsequent alcohol misuse.^7–10^ Three underlying theoretical mechanisms have been suggested to explain this phenomenon. (i) Alcohol consumption during adolescence disrupts important developmental processes, e.g. brain development, and thereby increases the risk of alcohol dependence.^11^ (ii) An early onset of alcohol use at least partly overlaps with an increased psychosocial vulnerability that increases the likelihood of engaging in multiple problem behaviours,^12^ in accordance with the ‘Problem Behavior Theory’.^13^ (iii) An early onset is just a marker of alcohol problems that derive from a common vulnerability, such as genetics.^7^ Delaying the age of onset has been considered as a major public health task in order to protect adolescents from the negative consequences of alcohol.^14^^,^^15^ However, a systematic review of longitudinal studies identified only five studies in this field with at least 3 years’ follow-up and outcomes measured in adulthood. Some evidence was found of a small but inconsistent effect that attenuated or disappeared when adjusting for confounders. Thus, the authors concluded that we do not yet know whether early onset leads to alcohol problems in adulthood.^16^

Another body of evidence suggests that drinking patterns in adolescence strongly predict alcohol consumption in adulthood.^17^ Given this evidence, some researchers highlight the potential long-term public health gains of the trend of decreased adolescent alcohol consumption.^18^^,^^19^ However, there is scarce empirical evidence regarding if these new generations of adolescents will continue with their modest alcohol consumption as they grow older or if they will ‘catch up’. To our knowledge, only three studies have examined whether the declined alcohol consumption among adolescents persists into adulthood, where two used repeated waves of cross-sectional surveys. Data from a nationally distributed health survey from 1981 to 2013 in Finland suggested no differences in alcohol consumption at age 18 years between more recent cohorts compared with older ones, despite a postponed age of onset.^20^ On the other hand, analyses of data from the National Drug Strategy Household Survey in Australia showed that adolescents seemed to partly ‘catch up’ with previous cohorts by early adulthood, but still had lower levels of consumption and risky drinking.^21^ The third study, however, used longitudinal data from the Household Income and Labour Dynamics in Australia survey from 2001 to 2016 and showed that more recent cohorts of 15-year-old adolescents compared to earlier cohorts, did catch up in their alcohol consumption at 24 years of age, but not enough, which indicates lower alcohol consumption in their 20s.^22^ However, this study compared drinking trajectories without considering possible confounders.

To our knowledge, this study is one of the first that uses longitudinal data from a birth cohort of people who were adolescents in the 2010s, when the decline in alcohol use among adolescents occurred, in order to examine subsequent alcohol outcomes of non-drinkers. In particular, the aim of the present study was to examine whether adolescents who had not used alcohol in ninth grade (14–15 years) drank less alcohol in their late teens (17–18 years) and young adulthood (20–21 years) than their drinking peers or if they caught up. Further, in contrast to the study of Callinan *et al*.,^22^ this study controls for possible confounders.

## Methods

### Participants

Data from a prospective cohort study, the Survey of Adolescent Life in Västmanland (SALVe cohort), were used. This cohort study follows all adolescents who were born in 1997 and 1999 in the Swedish region of Västmanland, a medium-sized region ∼90 km from the capital city Stockholm. In this study, only adolescents born in 1997 were included, since only 1.7% of those born in 1999 had used alcohol at baseline when they were 12–13 years old compared with 20.9% for those born in 1997. The cohort was first invited to participate in autumn 2012 (baseline) when they attended ninth grade (14–15 years). Of 2423 eligible students, 932 (38.46%) responded at baseline. In 2015, a 3-year follow-up was conducted when the responders attended 12th grade (late teens, 17–18 years). In autumn 2018, when participants were in young adulthood (20–21 years), they were assessed again in a 6-year follow-up. Of the 932 responders at baseline, 801 (85.94%) responded at the 3-year follow-up and 573 (61.48%) responded at the 6-year follow-up. However, seven responders at the 3-year follow-up and four at the 6-year follow-up had failed to answer the question on alcohol use at baseline and were therefore excluded. Thus, the final sample consisted of 794 participants (57.05% females) at the 3-year follow-up (late teens) and 569 participants (62.04% females) at the 6-year follow-up (young adulthood).

### Procedure

At baseline, participants were invited by post to participate in the study, sign a written consent form, and return a self-report questionnaire. At the 3- and 6-year follow-ups, a self-report questionnaire was sent to participants by post. The study was approved by the Ethical Review Board in Uppsala (Dnr. 2012/187).

### Measures

#### Alcohol outcomes

Alcohol outcomes included alcohol consumption and harmful alcohol use at the two follow-ups and alcohol use at baseline measured using the Alcohol Use Disorders Identification Test-Consumption (AUDIT-C).^23^ AUDIT-C consists of the three first consumption items of the full AUDIT. At the 3-year follow-up, an adolescent-modified version was used where one additional response option was added for questions 1 and 2 (the response ‘monthly or less’ was divided into ‘every other month or less’ and ‘about once a month’). Alcohol use at baseline was measured using the first item of the modified AUDIT-C, ‘How often during the past 12 months did you have a drink containing alcohol?’ with participants answering ‘never’ classified as non-drinkers and all others as alcohol users. In the 6-year follow-up, the AUDIT-C was used with slightly modified response options for the third question (‘How often did you have six or more drinks on one occasion?’) where ‘weekly’ was replaced with ‘2–4 times per month’, ‘daily or almost daily’ was replaced with ‘2–3 times per week’, and an additional response option was included: ‘4 or more times per week’. The threshold for harmful use at the 3-year follow-up was ≥8 for males and ≥7 for females, equivalent to the 75th percentile. The threshold was chosen to reflect the proportion of harmful use among 16- to 29-year-olds in the general population, which in 2016 was 25% when using an AUDIT score threshold of 6 for males and 5 for females.^24^ Similarly, the threshold for harmful use at the 6-year follow-up was ≥7 for males and ≥6 for females.

#### Confounders

Confounders included gender, country of birth, employment status of mother and father, externalizing problems, including symptoms of attention-deficit hyperactivity disorder (ADHD) and conduct problems. All confounders were measured at baseline except employment status of mother and father, which were measured at the 3-year follow-up. Country of birth was classified as Sweden, European country (participant or at least one of the parents born elsewhere in Europe or North America) or Country outside Europe (participant or at least one of the parents born outside Europe or North America). Employment status of mother and father was classified as Working/studying (including parental leave, housewife and other), On long-term sick leave or Unemployed. Symptoms of ADHD were measured with the Adult ADHD Self-Report Scale Adolescent version (ASRS-A).^25^ Conduct problems were measured using a 16-item questionnaire of delinquent and violent behaviour developed by Andershed *et al*.^26^

### Statistical analyses

First, in order to show the extent to which non-drinkers and alcohol users differed at baseline (14–15 years), confounders were compared using chi-squared tests for categorical variables and analysis of variance for continuous variables. Second, univariate differences in alcohol outcomes in late teens (17–18 years) and young adulthood (20–21 years) between non-drinkers and alcohol users at baseline were calculated with analysis of variance for alcohol consumption and chi-squared tests for harmful use. To avoid estimates of alcohol outcomes in the follow-ups being influenced by the number of non-drinkers, analyses were conducted on only participants who used alcohol at the follow-ups. Third, the importance of non-drinking at baseline for alcohol outcomes in late teens (17–18 years) and young adulthood (20–21 years) when adjusted for confounders was evaluated using multiple linear regression analysis for alcohol consumption and multiple logistic regression analysis for harmful alcohol use.

## Results

At baseline (14–15 years), the vast majority of participants (79.1%) were non-drinkers. Differences between non-drinkers and alcohol users at baseline are shown in Table 1. In contrast, only 17.5% of participants in their late teens (17–18 years) and 11.7% in young adulthood (20–21 years) were non-drinkers. Among non-drinkers at baseline, 78.3% had initiated alcohol consumption by their late teens (17–18 years) and 86.6% had done so by young adulthood (20–21 years). Further, a considerably larger proportion of non-drinkers at baseline remained non-drinkers in their late teens (17–18 years) as compared with the proportions transitioning from drinking to non-drinking, 21.7% vs. 2.4%. Corresponding numbers in young adulthood (20–21 years) were 13.4% who remained non-drinkers vs. 5.7% who transitioned from drinking to non-drinking.

**Table 1:** Differences between non-drinkers and alcohol users at baseline (except mother’s and father’s employment status, assessed at the 3-year follow-up)

	Non-drinkers	Alcohol users	Difference	*P*-value
Gender				
Male	83% (283)	17% (58)		
Female	76.2% (345)	23.8% (108)	5.492	0.019
Country of birth				
Sweden	78.9% (465)	21.1% (124)		
European country	74.2% (72)	25.8% (25)	2.910	0.233
Country outside Europe	84% (89)	16% (17)		
Mother’s employment status				
Working/studying	78.9% (585)	21.1% (156)		
On long-term sick leave	81% (17)	19% (4)	0.754	0.686
Unemployed	70.6% (12)	29.4% (5)		
Father’s employment status				
Working/studying	79.2% (590)	20.8% (155)		
On long-term sick leave	64.3% (9)	35.7% (5)	1.848	0.397
Unemployed	77.8% (14)	22.2% (4)		
Externalizing problems				
ADHD symptoms	20.08 (10.31)	25.21 (11.32)	30.965	˂0.001
Conduct problems	4.85 (5.10)	11.32 (8.87)	148.544	˂0.001

*Note*: Values are given as percentages (*N*) and mean values (standard deviations). Differences are expressed in chi-squared values for categorical variables and *F*-values for continuous variables.

Alcohol consumption outcomes in the late teens and young adulthood for baseline non-drinkers and alcohol users, respectively, are shown in Table 2, for males and females separately. In order to avoid bias in alcohol outcomes arising from non-drinkers at the follow-ups, these participants were excluded. Alcohol users at baseline had a significantly higher alcohol consumption in their late teens (17–18 years) with a mean difference of 2.12 AUDIT-C scores (*P* < 0.001) for males and 2.04 for females as compared with non-drinkers at baseline. No difference between baseline non-drinkers and alcohol users was found in young adulthood (20–21 years) for males, but a mean difference of 0.97 AUDIT-C scores (*P* < 0.001) was seen for females. Further, the proportion of harmful alcohol users in late teens (17–18 years) was approximately doubled among both male and female alcohol users at baseline compared with non-drinkers. In young adulthood (20–21 years), only a larger proportion of harmful alcohol use among female alcohol users compared to non-drinkers at baseline remained significant.

**Table 2:** Subsequent alcohol outcomes stratified by alcohol use at baseline

	Late teens (17–18 years)		Young adulthood (20–21 years)	
	AUDIT-C	Harmful use	AUDIT-C	Harmful use
Boys				
Non-drinker	5.37 (2.69)	27.7% (59)	4.88 (2.47)	26.9% (42)
Alcohol user	7.49 (2.56)***	50.9% (28)***	5.47 (2.77)	37.2% (13)
Girls				
Non-drinker	4.87 (2.30)	19.3% (52)	3.96 (2.09)	21.2% (49)
Alcohol user	6.91 (2.36)***	53.8% (57)***	4.93 (2.10)***	33.3% (27)*

*Note*: Only alcohol drinkers at follow-ups are included. Differences between non-drinkers and alcohol users were assessed with analysis of variance for AUDIT-C and chi-squared tests for harmful use.

* *P* ˂ 0.05,

** *P* ˂ 0.01,

*** *P* ˂ 0.001.

Effect sizes for the differences are shown in Table 3 and were calculated with linear and logistic regression analysis adjusted for covariates. Both univariate and multivariate associations were assessed. In the adjusted model, drinking alcohol at baseline (14–15 years) was associated with an increased alcohol consumption of 1.6 AUDIT-C scores (*P* < 0.001) and a 3-fold increased probability [odds ratio (OR) = 3.05, confidence interval (CI) = 2.00–4.65] of harmful alcohol use in the late teens (17–18 years). However, the effects of alcohol use at baseline on alcohol outcomes in young adulthood (20–21 years) decreased considerably and were no longer significant. *Post hoc* analyses (shown in Supplementary table S1) of both alcohol consumption and harmful alcohol use in young adulthood when each of the confounders was excluded from the analysis, revealed that the major decrease in effect from alcohol use occurred when conduct problems were included in the models. Therefore, an interaction term between alcohol use and conduct problems at baseline was added to the adjusted models. Although coefficients for the interaction were not significant, with *b* = −0.070 (*P* = 0.062) for alcohol consumption and OR = 0.94 (CI = 0.87–1.01) for harmful alcohol use, the close-to-significant interaction effects led us to conduct stratified analyses on one group lower than the median on conduct problems and one group higher than median. When adjusted for covariates (except conduct problems), alcohol use at baseline was associated with an increase of 1.553 AUDIT-C scores (*P* = 0.004) in young adulthood (20–21 years) in the group low on conduct problems as compared with 0.027 AUDIT-C scores (*P* = 0.330) in the group high on conduct problems. Similarly, alcohol use at baseline was associated with a higher probability (OR = 5.26, CI = 1.91–14.48) of harmful alcohol use in the group low on conduct problems as compared with that (OR = 0.88, CI = 0.48–1.82) in the group high on conduct problems.

**Table 3:** Regression coefficients (*b* and OR) for the associations between drinking status at baseline and subsequent alcohol outcomes, adjusted for covariates

	AUDIT-C		Harmful use	
	*b*	*P*-value	OR	95% CI
Late teens (17–18 years)				
Univariate association				
Alcohol use	1.890	˂0.001	3.75	2.57–5.45
Multivariate associations				
Alcohol use	1.579	˂0.001	3.05	2.00–4.65
Gender (female)	−0.576	˂0.001	0.81	0.55–1.19
Country of birth	−0.193	0.189	0.85	0.64–1.14
Mother’s employment status	−0.504	0.100	0.47	0.22–0.98
Father’s employment status	−0.499	0.107	0.57	0.28–1.14
ADHD symptoms	0.011	0.300	1.01	0.99–1.03
Conduct problems	0.057	0.001	1.04	1.01–1.08
Young adulthood (20–21 years)				
Univariate association				
Alcohol use	0.756	0.002	1.73	1.11–2.72
Multivariate associations				
Alcohol use	0.522	0.066	1.45	0.84–2.50
Gender (female)	−0.659	0.004	0.82	0.51–1.30
Country of birth	−0.430	0.014	0.74	0.50–1.09
Mother’s employment status	0.028	0.943	1.42	0.68–2.98
Father’s employment status	−0.228	0.478	0.67	0.31–1.44
ADHD symptoms	0.004	0.706	1.01	0.98–1.03
Conduct problems	0.048	0.023	1.02	0.98–1.06

*Note*: Only alcohol drinkers at follow-ups are included. Covariates in the analysis were treated as continuous variables.

## Discussion

The present study compared alcohol outcomes in the late teens (17–18 years) and young adulthood (20–21 years) between non-drinkers and alcohol users in ninth grade (14–15 years). Non-drinkers at baseline (14–15 years) had lower alcohol consumption than alcohol users and less probability of harmful alcohol use in their late teens (17–18 years), while the positive effect of non-drinking at baseline for alcohol consumption and harmful alcohol use in young adulthood (20–21 years) remained only for adolescents low on conduct problems.

First, the findings of this study may contradict one previously published study, which suggested that despite a postponed age of onset, alcohol use at age 18 years in more recent adolescent cohorts did not differ from that in older cohorts,^20^ indicating that the recent decline in adolescent alcohol consumption has limited effect on their alcohol consumption patterns at age 18 years. However, the latter study used aggregated survey data gathered bi-annually from adolescent populations in Finland to model how the proportion of alcohol use increased from age 12 to 18 years in more recent cohorts compared with older ones. Their approach differs from following a group of non-drinking adolescents longitudinally and evaluating their drinking at different ages.

Second, the finding that not drinking alcohol in ninth grade had a beneficial impact on subsequent alcohol consumption patterns which remained into the late teens (17–18 years) and young adulthood (20–21 years) for participants low on conduct problems is in line with evidence from McCambridge *et al*.^17^ who conducted a systematic review and concluded that drinking patterns in adolescence strongly predicted alcohol consumption in adulthood. Further, this finding may provide indicative support to the suggestion by Livingston *et al*.^18^^,^^19^ that the trend of decreased adolescent alcohol consumption may have public health gains, given the association between alcohol use in adolescence and adulthood and their findings that though younger adolescent cohorts seem to partly ‘catch up’ to older cohorts by early adulthood, they still have lower alcohol consumption and less risky drinking.^21^ Our finding of lower alcohol consumption in late teens and young adulthood, however, is in comparison with alcohol-drinking peers and not whether adolescents in the 2010s drink less than older cohorts of adolescents. Finally, this finding also supports the only study that used longitudinal data that suggested lower alcohol consumption at age 24 among more recent birth cohorts compared to earlier cohorts.^22^ However, this study extends the latter finding by controlling for relevant confounders.

Third, most non-drinkers at baseline (86.6%) had started to consume alcohol in young adulthood (20–21 years). Nevertheless, they had lower alcohol consumption and lower probability of harmful drinking compared with their alcohol-using peers in the late teens (17–18 years) and young adulthood (20–21 years) among those low on conduct problems. Thus, the mechanism behind the lower alcohol consumption among non-drinkers may be the delayed age of onset, which has been shown in some previous studies.^7–10^ Further, that the influence of alcohol consumption at baseline on alcohol consumption and harmful alcohol use in the late teens (17–18 years) remained when adjusted for confounders such as symptoms of ADHD and conduct problems may indicate that the effect of an early onset may be over and above psychosocial vulnerability that leads to engagement in multiple problem behaviours, at least in the late teens. This theory, the ‘Problem Behavior Theory’,^13^ has been suggested as an underlying mechanism for why an early onset constitutes a risk for alcohol problems.^7^ On the other hand, the major impact that conduct problems at baseline had on drinking pattern in young adulthood (20–21 years) supports the Problem Behavior Theory, which other studies have also observed. Maimaris and McCambridge^16^ found that rigorous control of confounders weakened or eliminated the effect of an early onset of alcohol, whereas the effect of an early onset of alcohol on heavy drinking in the mid-20s disappeared in a Norwegian longitudinal study when conduct problems were controlled for.^27^ It should be noted, however, that the present study did not measure when age of onset occurred. Therefore, this discussion should be interpreted with caution.

Fourth, the increased risk of elevated subsequent alcohol use that individuals with early conduct problems encounter is well known,^28^ which may explain our indicative finding that non-drinking adolescents high on conduct problems were the ones who ‘catch up’ their alcohol consumption in young adulthood (20–21 years). In contrast for those low on conduct problems, non-drinking adolescents at baseline had lower alcohol consumption and probability of harmful use in young adulthood than their alcohol-using peers.

Fifth, the findings of this study should be interpreted in the light of some major methodological limitations. One was the low response rate. The response rate at baseline was 38.46%, which indicates low generalizability. One consequence of the low response rate can be that those who participated in the study may be healthier than non-responders. The low proportion of alcohol users at baseline—where only 20.9% of the sample used alcohol compared with 56% of ninth graders in a national survey in 2012^29^—indicates that our sample was healthier. Thus, the healthier alcohol consumption at the follow-up among non-drinkers at baseline may have arisen due to selection bias. Another limitation was the small sample size. Although 569 responded to the follow-up in young adulthood (21–23 years), sub-grouping the sample into non-drinkers and alcohol users, and further into harmful use or not, decreased the statistical power. One consequence may be an increased risk of type II errors, which may be particularly relevant for data on effects in young adulthood (20–21 years). A third limitation was the limited follow-up period of 6 years, which may have produced temporary differences in alcohol outcomes—it might take a longer time for adolescents to ‘catch up’ their alcohol consumption. The non-significant effect on alcohol outcomes in young adulthood (20–21 years), and that only non-drinkers at baseline low on conduct problems displayed positive effects indicates that they may ‘catch up’ later. Finally, the additional *post hoc* analyses increase the risk of type 1 error. However, since the intention behind these additional analyses was exploratory to identify if any confounder had a large influence on the association between alcohol use and alcohol outcomes which could indicate a moderation effect, no Bonferroni correction was conducted. In an exploratory context when effect worthy of further study is examined, correction can be inappropriate.^30^

### Public health implications

This is one of the first studies to show that the decline in alcohol use that has occurred in the past decade among adolescents may be associated with healthier alcohol outcomes as the adolescents grow older, at least in the late teens and young adulthood for those low on conduct problems. This finding has promising implications for alcohol-related morbidity and mortality among the new generations of adolescents who largely do not use alcohol in their mid-teens and delay their alcohol onset. The study also supports prevention/interventions that aim to delay the age of alcohol onset.

## Conclusions

Adolescents in ninth grade (14–15 years) who do not use alcohol have lower alcohol consumption and less probability of harmful alcohol use in their late teens (17–18 years) and young adulthood (20–21 years) among those low on conduct problems, compared with their alcohol-using peers. This study is one of the first to suggest that the decline in alcohol use seen among adolescents in the past decade may result in a lower alcohol consumption as they grow older.

## Supplementary Material

ckad057_Supplementary_Data

## Data Availability

Data can be shared upon request to the corresponding author.
