# Supplementary material for: Do non-drinking youth drink less alcohol in young adulthood or do they catch up? Findings from a Swedish birth cohort
Source: Eur J Public Health. 2023 Apr 20;33(4):640–4. doi: 10.1093/eurpub/ckad057 (PMC10393482; doi:10.1093/eurpub/ckad057)
Supplement: ckad057_Supplementary_Data [file ckad057_supplementary_data.docx]

Table S1. Regression coefficients (b and OR) for the associations between drinking status at baseline and subsequent alcohol outcomes when each of the covariates (in brackets) were excluded from the model.

|  | AUDIT-C | |  | Harmful use | |
| --- | --- | --- | --- | --- | --- |
|  | *b* | *p* |  | *OR* | 95% CI |
| Late teens (17-18 years) |  |  |  |  |  |
| Multivariate associations |  |  |  |  |  |
| Alcohol use (Full model) | 1.579 | ˂ .001 |  | 3.05 | 2.00-4.65 |
| Alcohol use (Gender) | 1.463 | ˂ .001 |  | 2.91 | 1.93-4.40 |
| Alcohol use (Country of birth excluded) | 1.579 | ˂ .001 |  | 3.04 | 1.99-4.63 |
| Alcohol use (Mother’s employment status) | 1.569 | ˂ .001 |  | 2.97 | 1.95-4.51 |
| Alcohol use (Father’s employment status) | 1.593 | ˂ .001 |  | 3.09 | 2.03-4.70 |
| Alcohol use (ADHD symptoms) | 1.566 | ˂ .001 |  | 3.00 | 1.97-4.56 |
| Alcohol use (Conduct problems) | 1.889 | ˂ .001 |  | 3.71 | 2.50-5.51 |
|  |  |  |  |  |  |
| Young adulthood (20–21 years) |  |  |  |  |  |
| Multivariate associations |  |  |  |  |  |
| Alcohol use (Full model) | 0.522 | .066 |  | 1.45 | 0.84-2.50 |
| Alcohol use (Gender) | 0.376 | .180 |  | 1.38 | 0.11-2.36 |
| Alcohol use (Country of birth excluded) | 0.490 | .085 |  | 1.41 | 0.82-2.43 |
| Alcohol use (Mother’s employment status) | 0.517 | .067 |  | 1.46 | 0.85-2.51 |
| Alcohol use (Father’s employment status) | 0.531 | .060 |  | 1.50 | 0.87-2.57 |
| Alcohol use (ADHD symptoms) | 0.514 | .068 |  | 1.43 | 0.83-2.46 |
| Alcohol use (Conduct problems) | 0.802 | .002 |  | 1.65 | 1.01-2.68 |

Note. In young adulthood and in models without conduct problems, the coefficients for alcohol use at baseline were significant and dropped when conduct problems were included and became non-significant.
